# Surgery

**Published:** 1905-10-14

**Authors:** 


### Progress in Medicine and Surgery.

### SURGERY.

### FRACTURES.

(*Continued from page* 11.)

*Fracture of the Patella.*—During the past few years there has been an increasing tendency towards the open method of operative treatment of fractured patella. In an admirable short paper Mr. Hugh M. Rigby[12] gives his personal experience of 21 cases submitted to this operation in four years. It is not every surgeon, however, that will accept the gospel he preaches of wiring every fractured patella by this method unless some definite contra-indication is present. Indeed, among his cases there was one that ended in a disastrous result, namely, amputation of the thigh—an occurrence, of course, never met with in non-operative treatment of simple fracture. Again, Mr. Rigby rather over-estimates the disability in all cases of simple fracture treated by non-operative interference. Some of his cases of old fracture with wide separation showed great limitation of extension power, but it is well known that this is not always the case. Labourers, the subjects of long-standing fracture of the patella with separation of 5 inches and more between the fragments, have been known to follow their occupation as bricklayers, mounting a ladder with ease, sometimes carrying a hod on the shoulder. Mr. Rigby's arguments against the open and subcutaneous operations of wiring or suturing, such as Kochers' or Barker's methods, are quite conclusive:—(1) The wires are not permanent; they have to be taken out after three or four weeks; (2) *early* passive movements cannot be thoroughly carried out; (3) the fractured surfaces of the fragments cannot be satisfactorily freshened; (4) the blood and effusion in the joint remain untouched; (5) the torn tendinous expansions of the vasti are left untreated; (6) an exact coaptation of the fractured surfaces is out of the question, the common tilting of the lower fragment remaining partially uncorrected. His main argument for the open method and wiring is—(1) That the loss of time consequent upon the receipt of the injury is enormously reduced. A clear gain of about six months can be reckoned on in an average case. (2) That the operation ensures sound bony union with a perfect functional result. If there be much bruising or effusion in the joint he prefers to wait for a few days—he uses an electric drill to bore the holes, and two sutures of silver wire of medium thickness, but does not irrigate the joint with saline fluid, merely mopping out the blood-clot and serum with sterilised gauze. Importance is attached to the careful suturing of the lateral expansion of the torn quadriceps. Some surgeons in addition to this unite the lacerated tendon over the front of the patella. In comminuted or compound fracture operative treatment by wiring or suturing the fragment with silk or kangaroo tendon give excellent results. Dr. Rigby in every case leaves two small drainage tubes in for 48 hours. Massage and passive movement are commenced after 12 days.

*Epiphysial Beak of Tibia.*—To skiagraphy must be given the credit of bringing to light the occurrence of injuries to the smaller epiphysial processes. In the case of the epiphysis of the tubercle of the tibia, Schlatter, Ludloff, and others have shown by means of the *x*-ray that a second centre of ossification is frequently present at the lower part of the epiphysial beak. An injury, often slight or more often entirely overlooked, gives rise to detachment and in some instances to displacement of this centre. These injuries are frequently followed by what may be considered a distinctly new affection, viz. inflammation of the epiphysial beak of the tibia, which may be mistaken for traumatic arthritis in recent injuries or for pyogenic or tuberculous osteomyelitis or new growth in more chronic conditions. Dr. R. Winslow [13] has recorded one such case in a boy aged 14 in whom the affection was symmetrical. As the local condition did not subside after rest, fixation of the knee and various other means of treatment, an operation was performed and an area of soft and spongy bone covered by thickened periosteum removed from the front of the tibia. There was no suppuration, and cultures taken proved sterile. The case got rapidly well. In contrast to this, two cases of rarefying ostitis confined to the portion of the tibial tubercle developed from the diaphysis have been recorded by Mr. G. H. Makins,[14] after local injury, such as a violent contraction of the quadriceps extensor muscle, but which had been insufficient to cause separation and displacement of the tibial tubercle. The instances occurred in boys aged respectively 12 and 14 years, and were accompanied by pseudo-inflammatory signs, viz. local swelling, heat and tenderness in the soft parts overlying and surrounding the tubercle. By the *x*-ray the underlying bone of the diaphysis exhibited signs of rarefaction in an increased transparency.

*Oblique Fracture of the Tibia.*—Mr. E. H. Bennett is undoubtedly right when he states that fracture of the tibial shaft alone is rare as a result of indirect violence although common enough when the violence is direct. Oblique fractures of the central portion of the shaft without accompanying fracture of the fibula are rare. He relates [15] the following interesting case, the only one he had seen, of oblique fracture of the tibia alone from indirect violence. A girl, aged 15, jumped from a height of about 12 feet on to the ground and immediately fell backwards. She could not put any weight on the leg or attempt to walk. There were found to be present pain and loss of power in the limb but no crepitus, deformity, or any abnormal mobility, yet the *x*-ray revealed the presence of a very oblique fracture traversing almost 4 inches of the shaft of the tibia in its middle segment; its direction was from above and without, downwards and inwards. Union was perfect in the ordinary time.

*Fractures of the Astragalus and Os calcis.*—Again, in the foot, skiagraphy has been of the greatest benefit to the surgeon in proving the very frequent occurrence of fracture of the tarsal bones. Dr. D. M. Eisendrath [16] relates four cases of the os calcis and astragalus. These injuries may be produced in one of six different ways. 1. Compression-fractures, produced by the patient falling from a height and striking the ground, occur most frequently; the weight of the body is transmitted to the astragalus and os calcis, which are wedged in between the bones of the leg and are crushed or compressed, so that they must break. Such compression-fractures are frequently associated with fractures of the malleoli or with a dislocation of the astragalus. The plane of fracture may be horizontal, vertical, or oblique. There may be a simple division of the bone into two large fragments, or they may be so splintered as to be scarcely recognisable. 2. Fractures of the neck of the astragalus following sudden dorsal flexion of the foot are usually in the frontal plane. The anterior edge of the tibia impinging on the neck of the astragalus and thus cutting its way through. Such fractures are frequently associated with fracture of the inner malleolus. 3. The astragalus and os calcis may both be fractured by forcible supination or pronation of the foot; these are probably produced through traction on the interosseous ligaments, which have greater resisting power than the bones. 4. The great tuberosity of the os calcis is frequently torn off by sudden contraction of the gastrocnemius and soleus muscles attached to the tendo-Achillis. 5. The bones may be fractured by direct crushing violence, as in run-over accidents, such as tram-car. Here other of the tarsal bones are often involved. 6. Gunshot fractures. The clinical signs of greatest value in diagnosis are; palpation of a detached fragment, pes valgus or varus, examination by the *x*-ray and the history of the injury. Swelling, with obliteration of the normal depressions about the ankle is more marked than in sprains. Crepitus and abnormal mobility can rarely be elicited and lowering of the malleoli is seldom sufficiently marked to be of value. Shortening of the foot when found is of great value. As to treatment, in simple fractures without displacement the foot should be immobilised at right angles in a removable casing well padded round the ankle, and massage should be begun on the third or fourth day. The casing should be worn for eight weeks when the patient may gradually put his weight on the foot. The pain and discomfort caused by traumatic flat foot which frequently follows as in simple sprains can be relieved by a suitable steel sole inside the foot. In simple fractures with displacement if the fragment is in any danger of causing necrosis of the skin over it, it should be removed by open operation under strict asepsis. If either bone is so badly splintered as to render its retention impossible it may be removed without marked loss of function in the foot. Compound crushing and gun-shot fractures are treated by the same rules as in similar injuries elsewhere.

[12] Practitioner, May 1905, p. 604. [13] Annals of Surg., February 1905, p. 278. [14] Lancet, July 22, 1905, p. 213. [15] Dublin Jour. of Med. Sci., May 1905, p. 379. [16] Annals of Surg., March 1905, p. 363.

